# Cognitive Enrichment Through Emotion Regulation: A Model of Successful Cognitive Aging

**DOI:** 10.1093/geroni/igaa057.2053

**Published:** 2020-12-16

**Authors:** Niccole Nelson, Cindy Bergeman, Nathan Rose

**Affiliations:** University of Notre Dame, Notre Dame, Indiana, United States

## Abstract

Cognitive Enrichment Through Emotion Regulation (CENTER) is a theoretical framework of development that emphasizes the role of individuals finding their proverbial “centers” in shaping their cognitive aging trajectories. Within the CENTER framework, dynamic interactions between emotion regulatory processes that occur in real time (i.e., micro-level time), and global psychological well-being that develops over several years (i.e., macro-level time), aid in the optimization of cognitive aging. Indeed, by successfully regulating emotional reactions to stress in real time, which is supported by global psychological well-being, individuals will minimize their accumulation of allostatic load across the lifespan. Such minimization of allostatic load is key to optimizing cognitive aging through emotion regulation under the CENTER framework. CENTER will be motivated by fusing research on cognitive aging, emotion regulation, stress-and-coping, allostatic load, and psychological well-being.

